# Multisystem inflammatory syndrome in children (MIS-C) and “Near MIS-C”: A continuum?

**DOI:** 10.3389/fped.2022.988706

**Published:** 2023-01-05

**Authors:** Sarah Khafaja, Nour Youssef, Zeinab El Zein, Celina F. Boutros, Samer Bou Karroum, Nour Abdel-Halim, Rim Salameh, Daniella Hodroj, Nour El Meski, Oussama Nasrallah, Aram Bidikian, Ghassan Bou Saba, Mariam T. Arabi, Rima Hanna-Wakim, Ghassan S. Dbaibo

**Affiliations:** ^1^ Center for Infectious Diseases Research (CIDR) and WHO Collaborating Center for Reference and Research on Bacterial Pathogens, American University of Beirut, Beirut, Lebanon; ^2^ Division of Pediatric Infectious Diseases, Department of Pediatrics and Adolescent Medicine, American University of Beirut Medical Center, Beirut, Lebanon; ^3^ Department of Pediatrics and Adolescent Medicine, American University of Beirut Medical Center, Beirut, Lebanon; ^4^ Faculty of Medicine, American University of Beirut, Beirut, Lebanon; ^5^ Division of Pediatric Cardiology, Department of Pediatrics and Adolescent Medicine, American University of Beirut Medical Center, Beirut, Lebanon

**Keywords:** fever, COVID-19, SARS-CoV-2, multisystem inflammatory syndrome in children (MIS-C), Lebanon, children, adolescents

## Abstract

**Introduction:**

Reports of multisystem inflammatory syndrome in children (MIS-C), following severe acute respiratory syndrome coronavirus 2 (SARS-CoV-2) infection, have been increasing worldwide, with an incidence varying significantly across studies based on the definition used for the diagnosis. At our tertiary medical center in Lebanon, we encountered several cases that presented a diagnostic challenge because they mimicked MIS-C but did not meet the US Centers for Disease Control and Prevention (CDC) definition. We decided to review these cases and describe their features in comparison with cases that met the CDC criteria of MIS-C and those that had an alternative diagnosis.

**Methods:**

This is a retrospective chart review of subjects aged <19 years old admitted to the American University of Beirut Medical Center (AUBMC) between March 1, 2020, and May 31, 2021, with suspected or confirmed MIS-C, following documented COVID-19 infection, with sufficient or insufficient criteria for diagnosis. Subjects were classified into 3 groups: “MIS-C”, “Near MIS-C” and “Alternative Diagnosis”.

**Results:**

A total number of 29 subjects were included in our cohort. Fever was present in all subjects. In the MIS-C group, evidence for cardiovascular system involvement was the most common feature followed by the mucocutaneous and gastrointestinal systems. In the “Near MIS-C” and “Alternative Diagnosis” group, gastrointestinal symptoms were the most common with only one patient with cardiac abnormalities and none with coagulopathy. Subjects with typical MIS-C presentation had higher inflammatory markers when compared to subjects in the other groups. Almost all the subjects had positive IgG for SARS-CoV-2. Of the 29 subjects, the Royal College of Paediatrics and Child Health (RCPCH) case definition would have identified all suspected cases without an alternative diagnosis as MIS-C, whereas the World Health Organization (WHO) and the CDC definitions would have excluded 6 and 10 subjects, respectively.

**Conclusion:**

MIS-C presents a diagnostic challenge due to the nonspecific symptoms, lack of pathognomonic findings, and potentially fatal complications. More research is needed to fully understand its pathogenesis, clinical presentation spectrum, and diagnostic criteria. Based on our experience, we favor the hypothesis that MIS-C has a continuum of severity that necessitates revisiting and unifying the current definitions.

## Introduction

A novel coronavirus, the severe acute respiratory syndrome coronavirus 2 (SARS-CoV-2), was recognized in December 2019 in China following the emergence of unexplained severe lower respiratory infections in clusters of patients (1, 2). The World Health Organization (WHO) has declared the novel coronavirus (COVID-19) outbreak a global pandemic on March 11, 2020 (3). The COVID-19 pandemic continues to spread rapidly, presenting with a wide range of clinical manifestations from asymptomatic to severe acute respiratory distress syndrome, multiorgan failure and death (4). Initially, the pediatric patients were largely overlooked during the COVID-19 pandemic and were considered a low-risk population, as they accounted for less than 8% of the total cases, and the largest cohort with high morbidity and mortality was reported in the elderly (5–7). In late April 2020, reports from the United Kingdom emerged, describing children who required admission to intensive care units due to an unexplained multisystem inflammatory syndrome with features resembling Kawasaki disease and toxic shock syndrome (8). Subsequently, similarly affected children were reported across Europe and the United States, associated temporally and geographically with COVID-19 outbreaks (4). When an increase in the pediatric COVID-19 cases associated with hyperinflammation was described, the World Health Organization (WHO), the US Centers for Disease Control and Prevention (CDC), and the Royal College of Paediatrics and Child Health (RCPCH) developed overlapping definitions of the syndrome and named it multisystem inflammatory syndrome in children (MIS-C) or paediatric multisystem inflammatory syndrome—temporally associated with SARS-CoV-2 (PIMS-TS or PIMS) (8). These definitions have common elements, such as prolonged fever, multi-organ dysfunction, elevated inflammatory markers, and recent or current SARS-CoV-2 infection or exposure, however, they slightly differ in many other criteria (8–11).

The epidemiology of MIS-C remains unclear, however it appears to be a rare condition with an incidence <1% in children infected with SARS-CoV-2 (12). The mortality and morbidity in those patients differ significantly from the benign course of COVID-19 in children. The majority had previous SARS-CoV-2 infection or known exposure or serologic evidence of SARS-CoV-2, supporting the hypothesis that MIS-C represents an immune-mediated and dysregulated post-infectious inflammatory response possibly triggered by SARS-CoV-2 (13, 14).

Obviously, epidemiological data including incidence of MIS-C and the clinical findings varied significantly across studies, based on the definition used for diagnosis, with broader criteria leading to reports of a higher incidence (8, 15). Despite the risk of overdiagnosis, the true incidence of MIS-C may be significantly higher due to the lack of clinical awareness and specific diagnosis. Furthermore, diagnostic criteria were based on clinical manifestations in children hospitalized with severe disease, which may neglect mild cases (8).

As more cases are described in the literature, the clinical heterogeneity of the disease and its wide spectrum are being further understood. At present, our knowledge of this inflammatory syndrome is incomplete and the available results from published studies provide insufficient insights into the full epidemiological, clinical, immunological, and prognostic spectrum of MIS-C. Therefore, it is pivotal to undergo further studies in order to obtain a better definition of MIS-C, to optimize the characterization and the diagnostic criteria, to assess its true impact and to generate the best clinical and therapeutic approach, in addition to clarifying short term and long-term outcomes.

Following a wave of COVID-19 infections in Lebanon, we encountered several patients suspected to have MIS-C at our tertiary medical center in Lebanon over a 15-month period. Some of these patients presented a diagnostic challenge because they mimicked MIS-C but did not meet the CDC definition (9). We decided to review these cases and describe their features in comparison with cases that met the CDC criteria of MIS-C or those that had an alternative diagnosis.

## Materials and methods

### Study design

This is a retrospective chart review of patients with suspected or confirmed MIS-C, following current or recent SARS-CoV-2 infection or exposure to a person with suspected or confirmed COVID-19 infection, who had sufficient or insufficient criteria for diagnosis. The study was conducted at the American University of Beirut Medical Center (AUBMC), located in Beirut, Lebanon, and was approved by the institutional review board (IRB) at AUBMC (IRB ID: BIO- 2021- 0090). All patients were identified retrospectively, and their charts were reviewed through medical records by looking at the following ICD-10 codes for admission diagnosis: “Fever of other and unknown origin”, “fever” and “multisystem inflammatory disorder”. In addition, at least one of the authors on this paper was on the Pediatric Infectious Diseases (PID) service, which is consulted on cases suspected to have MIS-C, for the duration of the study period. Therefore, we used the PID service records to identify any patients that may have been missed by the hospital medical record review.

All children and adolescents <19 years of age, admitted to the hospital between March 1, 2020, to May 31, 2021 were included in the study if they had suspected MIS-C on admission. All included subjects had recent or past infection of SARS-CoV-2 confirmed by real-time reverse-transcriptase -polymerase -chain-reaction (RT-PCR) and/or serology. Notably, COVID-19 vaccines were not available for children and adolescents during the study period.

### Subject classification

Included subjects were categorized into the “MIS-C group” when they fulfilled the criteria for MIS-C CDC case definition. Subjects who were thought to have MIS-C upon presentation but did not fulfill the CDC criteria and had no alternative diagnosis, were categorized into the “Near MIS-C” group. The third group “Alternative Diagnosis” included subjects who were initially worked up for MIS-C but ended up with an alternative diagnosis.

In this study, categorization of subjects was based on the CDC case definition for MIS-C throughout the whole analysis, except for the first table of the analysis where subjects were classified according to the three most recognized definitions, the RCPCH, the WHO and the CDC.

### Definitions

As the COVID-19 pandemic evolved in different countries, the RCPCH, the WHO and the CDC had developed, based on a limited number of cases, different definitions for the multisystem inflammatory syndrome in children (Table 1) (9–11, 16).

**Table 1:** Case definitions for Multisystem inflammatory syndrome in children.

CDC^a^	WHO	RCPCH
An individual aged <21 years presenting with: • Fever >38.0 °C for ≥24 h or report of subjective fever lasting ≥24 h • Laboratory evidence of inflammation, Including, but not limited to, one or more of the following: an elevated CRP, ESR, fibrinogen, procalcitonin, d-dimer, ferritin, LDH, or IL-6, elevated neutrophils, reduced lymphocytes, and low albumin • Evidence of clinically severe illness requiring hospitalization • Multisystem (≥2) organ involvement (cardiac, renal, respiratory, hematologic, gastrointestinal, dermatologic, or neurological) **AND** No alternative plausible diagnoses **AND** Positive for current or recent SARS-CoV-2 infection by RT-PCR, serology, or antigen test; or exposure to a suspected or confirmed COVID-19 case within the 4 weeks prior to the onset of symptoms	Children and adolescents 0–19 years of age with fever ≥3 days **AND** two of the following: 1. Rash or bilateral non-purulent conjunctivitis or muco-cutaneous inflammation signs (oral, hands or feet). 2. Hypotension or shock. 3. Features of myocardial dysfunction, pericarditis, valvulitis, or coronary abnormalities (including ECHO findings or elevated Troponin/NT-proBNP), 4. Evidence of coagulopathy (by PT, PTT, elevated d-Dimers). 5. Acute gastrointestinal problems (diarrhoea, vomiting, or abdominal pain). **AND** Elevated markers of inflammation such as ESR, C-reactive protein, or procalcitonin. **AND** No other obvious microbial cause of inflammation, including bacterial sepsis, staphylococcal or streptococcal shock syndromes. **AND** Evidence of COVID-19 (RT-PCR, antigen test or serology positive), or likely contact with patients with COVID-19.	A child presenting with persistent fever, inflammation (neutrophilia, elevated CRP and lymphopenia) and evidence of single or multi-organ dysfunction (shock, cardiac, respiratory, renal, gastrointestinal or neurological disorder) with additional features (see listed in Appendix 1). This may include children fulfilling full or partial criteria for Kawasaki disease^b^. Exclusion of any other microbial cause, including bacterial sepsis, staphylococcal or streptococcal shock syndromes, infections associated with myocarditis such as enterovirus (waiting for results of these investigations should not delay seeking expert advice). SARS-CoV-2 PCR testing may be positive or negative

CRP, C-reactive protein; ESR, erythrocyte sedimentation rate; LDH, lactic acid dehydrogenase; IL-6, interleukin 6; RT-PCR, reverse transcriptase–polymerase chain reaction; ECHO, echocardiography; NT-proBNP, N-terminal pro–B-type natriuretic peptide; PT, prothrombin time; PTT, activated partial thromboplastin time.

^a^ Additional comments: Some individuals may fulfill full or partial criteria for Kawasaki disease^#^ but should be reported if they meet the case definition for MIS-C. Consider MIS-C in any pediatric death with evidence of SARS-CoV-2 infection.

^b^ Criteria for Kawasaki disease include persistent fever and 4 of 5 principal clinical features: erythema and cracking of lips, strawberry tongue, and/or erythema of oral and pharyngeal mucosa; bilateral bulbar conjunctival injection without exudate; rash (maculopapular, diffuse erythroderma); erythema and edema of the hands and feet and/or periungual desquamation; and cervical lymphadenopathy.

Different patterns of clinical presentation and organ system involvement were present in our subjects and ranged from mild to severe forms. Many subjects had mild upper or lower respiratory symptoms such as sore throat, nasal congestion, rhinorrhea or cough, or very mild mucocutaneous symptoms which were not clinically suggestive of significant organ involvement. We described organ system involvement based on the symptoms, clinical findings, and laboratory results (17).

### Data collection

The data collection was performed by the study team at the Center of Infectious Diseases Research Center (CIDR). Variables recorded from admission to discharge were extracted from the clinical records assuring anonymity and confidentiality. Variables included demographics, comorbidities, history of COVID-19 infection or exposure to a COVID-19 positive person, presenting signs and symptoms, associated comorbidities, physical examination findings, laboratory tests, imaging reports, viral testing with SARS-CoV-2 RT-PCR and serology tests, clinical course in the hospital, management, and outcomes.

### Statistical analysis

Descriptive analyses were performed using numbers and percentages for qualitative variables and means with standard deviation (SD) or median with range for continuous variables. All statistical analyses were performed with the use of the Statistical Package for Social Sciences (SPSS) program, version 25.0 for Windows (Armonk, NY, IBM Corp).

## Results

In the 15-month study period, a total number of 29 subjects who presented with a clinical picture suspicious of MIS-C were included in our cohort, with a median age of 5 years.

Based on the RCPCH definition, 21 subjects fit the definition of PIMS (MIS-C), but when the WHO and the CDC case definitions were used, only 15 and 11 subjects, respectively, fit the MIS-C definition (Table 2). Most of our subjects were previously healthy; however, 24.1% of them had preexisting medical conditions and comorbidities including hematological malignancies, sickle cell disease, thalassemia, hemophagocytic lymphohistiocytosis (HLH) or obesity. The “Alternative Diagnosis” group included 8 subjects whose workup showed other etiologies including drug-induced pericarditis, salmonella or rotavirus gastroenteritis, adenovirus or rhinovirus/enterovirus infection documented by multiplex PCR respiratory panel, urinary tract infection (UTI), or brucellosis. Overall, there was a male predominance (69%), which was also noted in both MIS-C and “Near MIS-C” groups (81.8% and 80% respectively). Almost 90% of the subjects had history of recent SARS-CoV-2 infection by RT-PCR or exposure to a suspected or confirmed COVID-19-infected person within the 2 to 8 weeks prior to presentation (Table 3).

**Table 2:** Cases classification based on different definitions.

	CDC, *n* (%) *N* = 29	WHO, *n* (%) *N* = 29	RCPCH, *n* (%) *N* = 29
MIS-C	11 (37.9)	15 (51.7)	21 (72.4)
Near MIS-C	10 (34.5)	6 (20.7)	0 (0.0)
Alternative diagnoses	8 (27.6)	8 (27.6)	8 (27.6)

**Table 3:** Demographic and clinical characteristics of the patients.

Characteristics	Total, *n* (%) *N* = 29	MISC, *n* (%) *N* = 11	Near-MISC, *n* (%) *N* = 10	Alternative diagnoses, *n* (%) *N* = 8^a^
Median age in years (± SD)	5.0 (1.0–17.0)	8.0 (3.0–17.0)	4.0 (1.0–17.0)	3.0 (1.16–13.0)
Gender				
* Male*	20 (69.0)	9 (81.8)	8 (80.0)	3 (37.5)
* Female*	9 (31.0)	2 (18.2)	2 (20.0)	5 (62.5)
Comorbidities^b^	7 (24.1)	4 (36.4)	2 (20.0)	1 (12.5)
History of recent SARS-CoV-2 infection by RT-PCR or exposure to a suspected or confirmed COVID-19 person				
* No*	3 (10.3)	1 (9.1)	1 (10.0)	1 (12.5)
* Yes*	26 (89.7)	10 (90.9)	9 (90.0)	7 (87.5)

^a^ The alternative diagnoses found in our subjects were pericarditis, salmonella or rotavirus gastroenteritis, adenovirus or rhinovirus/enterovirus infection, urinary tract infection, or brucellosis.

^b^ Comorbidities included hematologic malignancies, Sickle cell disease, Thalassemia trait, Hemophagocytic lymphohistiocytosis or obesity.

Table 4 summarizes the clinical presentation of the subjects. Fever was present in all subjects with a difference in the median duration between the MIS-C group (6 days), the near-MIS-C group (2 days) and the alternative diagnosis group (4 days). Gastrointestinal symptoms were prevalent in the 3 groups. Respiratory symptoms were most encountered in the MIS-C group. When comparing the 3 groups, mucocutaneous involvement was reported in 81.8% of subjects with MIS-C while only 20% and 37.5% of the subjects in the “Near MIS-C” group and “Alternative Diagnosis” group, respectively, had conjunctivitis, cracked lips, strawberry tongue or a rash reported during illness.

**Table 4:** Clinical characteristics based on organ system involvement.

Organ system involvement	MISC, *n* (%) *N* = 11	Near-MISC, *n* (%) *N* = 10	Alternative diagnoses, *n* (%) *N* = 8
Respiratory involvement^a^	4 (36.4)	0 (0.0)	2 (25.0)
Gastrointestinal involvement	8 (72.7)	7 (70.0)	6 (75.0)
Mucocutaneous involvement	9 (81.8)	2 (20.0)	3 (37.5)
Cardiovascular involvement^b^	9 (90.0)	0 (0.0)	1 (12.5)
Hematological involvement	7 (63.6)	0 (0.0)	0 (0.0)

^a^ 6 patients did not have a chest x-Ray or Computed tomography (CT) of the chest performed as they did not have any significant respiratory symptom; one from the MISC group, 2 from the Near-MISC group, and 3 patients from the “Alternative diagnoses” group.

^b^ 4 patients did not undergo echocardiography, one from the MISC group and 3 from the Near-MISC group.

Out of 23 subjects who underwent imaging (chest x-ray or chest CT-scan), only 6 subjects had evidence of radiologically confirmed pneumonia, of which 4 subjects belonged to the MIS-C group. Echocardiography was performed in 25 subjects, where 9 out of 10 subjects in the MIS-C group had positive findings including 55.5% with reduced ejection fraction. One of these subjects had severe biventricular dysfunction (ejection fraction <20%) with increased pulmonary artery pressure requiring admission to the pediatric intensive care unit (ICU) and the use of vasopressors. Coronary artery dilation and pericardial effusion were noted, respectively, in one and 4 subjects in the MIS-C group. One subject from the “Alternative Diagnosis” group had moderate pericardial effusion. This subject, known to have hemophagocytic lymphohistiocytosis status four-months post hematopoietic stem cell transplant, showed typical findings of pericarditis for which he received steroids and non-steroidal anti-inflammatory agents. None of the subjects in the “Near MIS-C” group had cardiac involvement. Two subjects with MIS-C developed pleural effusion, whereas none of the subjects with “Near MIS-C” did. None of the subjects had neurological involvement.

Laboratory parameters were reviewed as shown in Table 5. Compared with the subjects with “Near MIS-C” and those with alternative diagnoses, subjects with typical MIS-C presentation had higher leukocytosis, platelet counts and acute inflammatory markers, specifically C-reactive protein (CRP), erythrocyte sedimentation rate (ESR), ferritin and D-dimer (Table 5). Elevated troponin and pro-B-type natriuretic peptide (Pro-BNP) levels were mainly detected in the MIS-C group with mean values that were significantly higher than the values reported for the remaining subjects. Among the subjects in the “Near MIS-C” and the alternative diagnoses groups who were serologically tested for SARS-CoV-2, 100% had positive serology test results, mainly IgG, with or without positive RT-PCR test results at one point during their illness whether prior to their presentation or concomitantly. As for the MIS-C group, out of the 11 tested subjects, SARS-CoV-2 IgG was detected in 10 subjects. The remaining subject had positive SARS-CoV-2 IgM and negative IgG as he developed MIS-C around 2 weeks after infection and therefore SARS-CoV-2 IgG did not rise yet.

**Table 5:** Laboratory tests.

Laboratory results	MISC, mean/ (number of tested patients) (±SD)	Near-MISC, mean/ (number of tested patients) (±SD)	Alternative diagnoses, mean/ (number of tested patients) (±SD)
Blood cell counts			
White blood cells (/cu.mm) (*N* = 29)	19,271/11 (±12,058)	9,410/10 (±4,860)	11,975/8 (±4,072)
Platelets (/cu.mm) (*N* = 29)	591,909/11 (±307,002)	291,800/10 (±86,672)	372,437/8 (±235,935)
Inflammatory markers			
CRP (mg/L) (*N *= 29)	176.6/11 (±95.6)	59.4/10 (±45.4)	95.3/8 (±118.2)
ESR (mm/hr) (*N* = 14)	71.4/7 (±34.6)	34.7/3 (±8.3)	35.5/4 (±15.5)
Procalcitonin (ng/ml) (*N* = 8)	6.4/7 (±3.6)	—	0.6/1
Ferritin (ng/ml) (*N* = 28)	909.6/11 (±1,040.8)	557.7/9 (±1,139.1)	137.6/8 (±56.2)
Interleukin-6 (pg/ml) (*N* = 4)	112.7/4 (±153.1)	—	—
Fibrinogen (g/L) (*N* = 25)	4.5/11 (±1.9)	3.6/8 (±0.9)	3.8/6 (±1.5)
D-Dimer (ng/ml) (*N* = 29)	3,038.9/11 (±1,623.3)	661.0/10 (±789.1)	731.7/8 (±287.5)
Cardiac markers			
Pro-BNP (pg/ml) (*N* = 16)	7,507.6/10 (±7,625.0)	268.0/2 (±66.5)	978.0/4 (±1,396.5)
Troponin T (ng/ml) (*N* = 29)	0.055 (±0.069)	0.005 (±0.002)	0.007 (±0.008)
	MISC, n/number of tested patients (%)	Near-MISC, n/number of tested patients (%)	Alternative diagnoses, n/number of tested patients (%)
SARS-CoV2 RT-PCR at presentation (*N* = 29)			
Negative	7/11 (63.6)	9/10 (90.0)	6 (75.0)
Positive	4/11 (36.4)	1/10 (10.0)	2 (25.0)
COVID IgM (*N* = 26)^a^			
Negative	9/10 (90.0)	8/8 (100)	8/8 (100)
Positive	1/10 (10.0)^b^	0/8 (0.0)	0/8 (0.0)
COVID IgG (*N* = 26)^a^			
Negative	1/10 (10.0)^b^	0/8 (0.0)	0/8 (0.0)
Positive	9/10 (90.0)	8/8 (100)	8/8 (100)

^a^ COVID antibodies were not tested in 3 patients, one patient who had acute COVID infection from the MISC group and 2 others from the Near-MISC group.

^b^ This patient had acute COVID infection, he developed MIS-C almost 11 days after infection.

The outcome and management of patients are shown in Table 6.

**Table 6:** Complications, outcome and management.

Complications, Outcome and Management	MISC, *n* (%) *N* = 11	Near-MISC, *n* (%) *N* = 10	Alternative diagnoses, *n* (%) *N* = 8
ICU admission	3 (27.3)	0 (0.0)	0 (0.0)
Length of stay (in days), median (range)	8.0 (1.0–22.0)	3.0 (1.0–5.0)	5.0 (3.0–19.0)
Treatment (*N* = 29)			
*Antibiotic therapy*	10 (90.9)	3 (30.0)	4 (50.0)
*IVIG*^a^	11 (100)	0 (0.0)	1 (12.5)
*Steroids*	9 (81.8)	1 (10.0)	1 (12.5)
*Aspirin*	10 (90.9)	2 (20.0)	1 (12.5)
*Enoxaparin*	7 (63.6)	1 (10.0)	0 (0.0)
*Vasopressors*	3 (27.3)	0 (0.0)	0 (0.0)

^a^ IVIG, intravenous immunoglobulins.

None of the 29 subjects required mechanical ventilation or extracorporeal membrane oxygenation. On admission, empiric intravenous broad-spectrum antibiotics were initiated in 91% of the subjects with MIS-C vs. 30% of the subjects with “Near MIS-C”. Blood and urine cultures were negative for all subjects. Upon presentation, 3 subjects in the MIS-C group presented with hypotension and/or cardiogenic shock requiring ICU admission and inotropic support. In the MIS-C group, all subjects (100%) were treated with intravenous immunoglobulins (IVIG), 81.8% received methylprednisolone, which was later shifted to oral prednisolone and tapered over 3 to 4 weeks. None of the subjects in the two other groups received IVIG nor vasopressors. Enoxaparin was given to 63.6% of subjects with MIS-C who had severe ventricular dysfunction, dilated coronary arteries, or markedly elevated D-dimer level. The only subject in the “Near MIS-C” group who received enoxaparin had a hematological malignancy and markedly elevated D-dimer level. No mortality was documented, all subjects recovered.

### Illustrative cases

**Figure 1:**
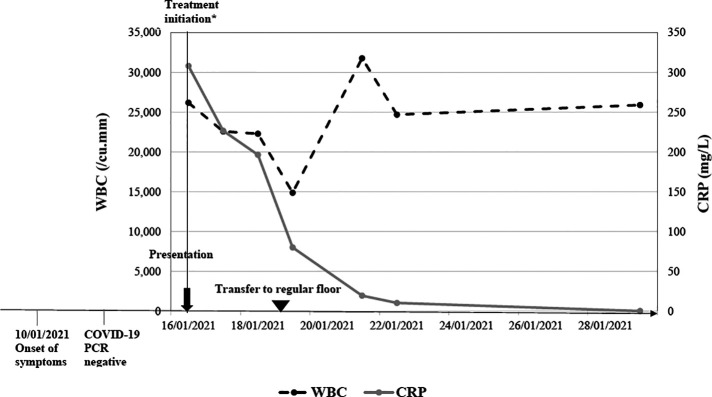
Clinical course of a 4-year-old with confirmed MIS-C. *Admission to Pediatric Intensive care unit and treatment administration including IVIG, steroids, aspirin and antibiotics.

**Figure 2:**
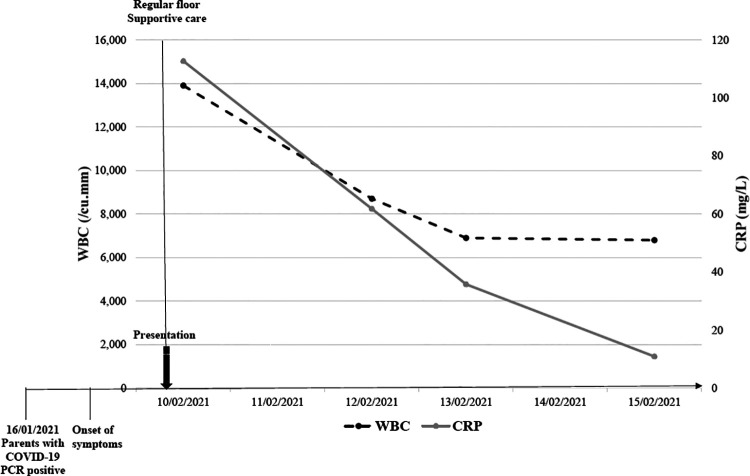
Clinical course of a patient with near MIS-C.

The clinical course of two representative subjects is shown in Figures 1, 2, respectively. The first subject is a 4-year-old patient with thalassemia trait who returned from a trip to Turkey 3 weeks prior to presentation. He presented to our center with high-grade fever of 6 days duration, nasal congestion, and rash. Two nasopharyngeal RT-PCR tests for SARS-CoV-2 in the preceding few days were negative. On physical examination, he had bilateral nonexudative conjunctivitis with limbic sparing and progressive erythematous maculopapular blanching rash over his upper and lower extremities. Laboratory work up was significant for leukocytosis (26,000/mm^3^) with neutrophil predominance, microcytic anemia, low albumin, prolonged prothrombin time and International Normalized Ratio (INR), and elevated inflammatory markers including CRP, procalcitonin, ferritin and D-dimer. His SARS-CoV-2 serology yielded a positive IgG indicating a previous infection. Echocardiography showed mildly reduced ejection fraction, prominent coronary arteries, as well as mild tricuspid and mitral regurgitation. He was initially hemodynamically unstable requiring admission to the ICU and inotropic support. He was started empirically on broad spectrum antibiotics (vancomycin and ceftriaxone). The patient was diagnosed with MIS-C based on the CDC case definition and received IVIG, steroids and low-dose aspirin.

The second subject is a 12-month-old boy who presented to the emergency department with a 2-day history of high-grade fever, associated with vomiting, decreased oral intake and urine output. His physical examination was remarkable for pharyngeal exudates, but no cervical adenopathy. The patient's parents had COVID-19 infection around 3 weeks prior to the onset of his symptoms, during which the patient had one episode of high-grade fever and diarrhea but was not tested. COVID-19 RT-PCR done on admission was negative while SARS-CoV-2 serology yielded a positive IgG and negative IgM. He had a WBC count of 13,900 /mm^3^ and CRP level of 112.7 mg/L. He was admitted to the pediatric unit for intravenous hydration and was started on aspirin as his echocardiography revealed prominent distal left anterior descending (LAD) coronary artery, with good biventricular function, and no pericardial effusion. This patient did not meet the full criteria for MIS-C and therefore was considered as a case of “Near MIS-C”. It is remarkable to mention that white count, platelet count, and inflammatory markers improved significantly at two, five, and twelve days later. In addition, the prominent distal LAD improved gradually on follow up echocardiography evaluations performed at one, three, and five months. However, it is worth mentioning that no further investigations to rule out a possible viral etiology were done since the patient clinically improved within two days without substantial intervention and that the initial presentation did not qualify for the diagnosis of Kawasaki disease (only 2 days of fever, no lymphadenopathy, mucositis, rash and never developed desquamation, or laboratory findings of thrombocytosis or hypoalbuminemia).

## Discussion

In the current series of patients with, or suspected to have, MIS-C, fever was present in all subjects including those from the “Near MIS-C” and “Alternative Diagnosis” groups. Cardiovascular system involvement was the most reported in our MIS-C group followed by mucocutaneous and gastrointestinal systems involvement. A recent systematic review also showed that cardiovascular and gastrointestinal symptoms were most commonly seen in MIS-C patients (15), in addition an Italian single-center study reported that 81% of their MIS-C patients had cardiac involvement, confirming that the heart along with the gastrointestinal system are most involved in MIS-C patients (18). Gastrointestinal symptoms were similarly encountered in the “Near MIS-C” and “Alternative Diagnosis” group but none had clinical evidence of coagulopathy or cardiac abnormalities except for one patient who was suspected to have cyclosporin-induced pericarditis. Hence, subjects with “Near MIS-C” did not meet the CDC case definition criteria but were hospitalized for close monitoring lest they develop additional organ involvement after their initial presentation.

MIS-C presents a diagnostic challenge for the physician due to the nonspecific early symptoms, lack of pathognomonic findings, and potentially fatal complications. Our understanding of the pathogenesis of this syndrome is evolving but remains limited. Whereas genetic predisposition to developing severe MIS-C has been demonstrated (19), the true triggers for the severe inflammatory response seen in otherwise healthy individuals with MIS-C and characterized by increased plasma cytokine levels including IL-6, IL-8, IFNγ, IL-17, TNFα, and IL-10 and the multilineage immune cell activation remain unknown (20–24). A specific immunopathologic signature and association with HLA alleles were identified recently that may pave the way for the development of more specific biomarkers (25). Importantly, the magnitude of the inflammatory response in MIS-C correlates with disease severity (23) and this raises the possibility that this syndrome is actually a continuum of clinical severity rather than an all-or-none response as the current definitions seem to imply.

In the present study, we compared the RCPCH, the WHO and the CDC case definitions (9–11). We found that the RCPCH definition was more inclusive and identified all subjects with MIS-C and “Near MIS-C” and excluded those who had features of MIS-C but were found to have an alternative diagnosis. The WHO and the CDC definitions are more stringent concerning clinical manifestations and the relationship with SARS-CoV-2 infection. In our case series, the WHO definition identified more subjects in the MIS-C group compared to the CDC definition, with few subjects whom we classified as “Near MIS-C” as they did not present multisystem dysfunction or did not have persistent fever for ≥3 days as per the WHO definition. In contrast to that of the WHO, the CDC definition excluded few subjects of those included in the MIS-C group based on the WHO definition, all failing to achieve the CDC criterion concerning the multi-organ (≥2 organ systems) dysfunction. Similarly, in a systematic review, Hoste et al. observed that although the RCPCH definition included all cases, nevertheless both the CDC and WHO case definitions were more selective, requiring multisystem involvement and a proven association with SARS-CoV-2, with the WHO definition including 97% of cases and the CDC definition 62% (15). Clearly a more precise definition for MIS-C based on a better understanding of the pathogenesis and the identification of more specific clinical and laboratory markers is needed.

The dynamic nature of this syndrome presents a further challenge to the physician. In the literature, several cases were reported to present with incomplete criteria initially but to later develop typical findings including cardiac involvement (16, 19). This has led us to hospitalize patients when the diagnosis of MIS-C was highly suspected, to provide close monitoring and rapid intervention when needed. This is further confounded by the frequent presence of another infection at the time of presentation. All the current definitions include the lack of “alternative plausible diagnoses (CDC)” (9), “other obvious microbial cause of inflammation (WHO)” (11), or “any other microbial cause (RCPCH)” (10). In our series, eight subjects ended up with an alternative diagnosis: drug-induced pericarditis, UTI, respiratory tract infections caused by adenovirus or rhinovirus/enterovirus, or infection with brucella, salmonella, or rotavirus. In these cases, the suspicion of MIS-C arose due to the temporal association with COVID-19 infection or exposure and the presence of commensurate clinical and laboratory findings including elevated inflammatory markers and D-dimers. In this regard, several questions present themselves for treating physicians: (1) how extensively should they work up the patient looking for an alternative diagnosis? (2) once identified, is the alternative diagnosis sufficient to explain the clinical presentation? (3) are the laboratory findings in keeping with the alternative diagnosis, e.g., does a rhinoviral infection explain the significantly elevated D-dimer level? Finally, (4) does the identification of an alternative diagnosis rule out the possibility of concomitant MIS-C? These questions present dilemmas for the treating physician who must decide on further treatment but has no discernible answers. An extensive work up to exclude a microbial infection may be necessary to rule out the majority of the alternative diagnoses, e.g., multiplex PCR respiratory and gastrointestinal panels that may not be readily available at most hospitals especially in underprivileged locations. Moreover, these additional tests present a significant economic burden on the healthcare system. On the other hand, not performing the extensive work up may lead to overdiagnosis of MIS-C as patients may satisfy all the criteria in the absence of an alternative diagnosis as reported previously (26).

Whether the identification of an alternative diagnosis is sufficient to explain the clinical and laboratory findings is not always straightforward. For example, all our subjects demonstrated elevation in inflammatory markers specifically CRP, ESR and D-dimer, which are nonspecific and overlap with many other infectious and inflammatory diseases (27). In the pre-COVID-19 era, and before the emergence of MIS-C, obtaining laboratory values such as ferritin and D-dimer for evaluation of febrile children was uncommon. In pediatric patients, D-dimer is requested for a select group of diagnoses including pulmonary embolism, disseminated intravascular coagulation, or hyperthrombotic states (28), however this is not the practice for other causes such as rhinovirus, rotavirus, or salmonella infections. For that reason, expected values for such tests in children with common febrile illnesses remain unknown (27). This is illustrated in the current series where we could not be certain whether the alternative diagnoses found in some of our subjects were associated intrinsically with elevated D-dimer or this finding was due to concomitant MIS-C or a recent COVID-19 infection. Not performing the D-dimer level doesn't seem to be a viable option as an increased level is sometimes the only clue for MIS-C and coronary artery dilatation (29). Thus, trending these inflammatory markers in subjects with suspected MIS-C, even in the presence of an alternative diagnosis, may help identify concomitant diagnoses of MIS-C, prevent disease progression, and multi-organ involvement (30).

Clearly, more research is needed to fully understand the pathogenesis of MIS-C and to fully appreciate its clinical presentation spectrum. Based on our experience reported in the current series, we favor the hypothesis that MIS-C has a continuum of severity where the most severe cases present as Kawasaki disease-like picture or shock/myocarditis and the less severe cases present with various degrees of fever and hyperinflammation. The current study is limited by its retrospective nature from a single center. As a result, not all cases suspected to have MIS-C were worked up to the same extent. Thus, larger prospective studies are required to verify this hypothesis to provide unambiguous answers. For example, current databases recruiting MIS-C patients (31–33) should expand to include subjects with “Near MIS-C” and even those with an alternative diagnosis that may not fully explain all the clinical and laboratory findings. This is going to be more important as the pandemic wanes and the disease becomes more sporadic presenting an even more difficult diagnostic challenge for treating physicians.

## Data Availability

The datasets presented in this article are not readily available because of privacy and ethical restrictions. Requests to access the datasets should be directed to the corresponding author.

## References

[1] Huang C Wang Y Li X Ren L Zhao J Hu Y Clinical features of patients infected with 2019 novel coronavirus in Wuhan, China. Lancet. (2020) 395(10223):497–506. 10.1016/S0140-6736(20)30183-5 31986264 PMC7159299

[2] Zhu N Zhang D Wang W Li X Yang B Song J A novel coronavirus from patients with pneumonia in China, 2019. N Engl J Med. (2020) 382(8):727–33. 10.1056/NEJMoa2001017 31978945 PMC7092803

[3] Cucinotta D Vanelli M. WHO Declares COVID-19 a pandemic. Acta Biomed. (2020) 91(1):157–60. 10.23750/abm.v91i1.9397 32191675 PMC7569573

[4] Rodriguez-Morales AJ Cardona-Ospina JA Gutiérrez-Ocampo E Villamizar-Peña R Holguin-Rivera Y Escalera-Antezana JP Clinical, laboratory and imaging features of COVID-19: a systematic review and meta-analysis. Travel Med Infect Dis. (2020) 34:101623. 10.1016/j.tmaid.2020.101623 32179124 PMC7102608

[5] Toraih EA Hussein MH Elshazli RM Kline A Munshi R Sultana N Multisystem inflammatory syndrome in pediatric COVID-19 patients: a meta-analysis. World J Pediatr. (2021) 17(2):141–51. 10.1007/s12519-021-00419-y 33608839 PMC7895741

[6] Garg S Kim L Whitaker M O’Halloran A Cummings C Holstein R Hospitalization rates and characteristics of patients hospitalized with laboratory-confirmed coronavirus disease 2019 - COVID-NET, 14 states, March 1–30, 2020. MMWR Morb Mortal Wkly Rep. (2020) 69(15):458–64. 10.15585/mmwr.mm6915e3 32298251 PMC7755063

[7] Jiang L Tang K Levin M Irfan O Morris SK Wilson K COVID-19 and multisystem inflammatory syndrome in children and adolescents. Lancet Infect Dis. (2020) 20(11):e276–88. 10.1016/S1473-3099(20)30651-4 32818434 PMC7431129

[8] Esposito S Principi N. Multisystem inflammatory syndrome in children related to SARS-CoV-2. Paediatr Drugs. (2021) 23(2):119–29. 10.1007/s40272-020-00435-x 33479801 PMC7819738

[9] CDC. Multisystem Inflammatory Syndrome (MIS) (2021). Available from: https://www.cdc.gov/mis/mis-c/hcp/index.html (Accessed May 11, 2022).

[10] Health RCoPaC. Paediatric multisystem inflammatory syndrome temporally associated with COVID-19 (PIMS) - guidance for clinicians (2020). Available from: https://www.rcpch.ac.uk/resources/paediatric-multisystem-inflammatory-syndrome-temporally-associated-covid-19-pims-guidance (Accessed May11, 2022).

[11] Organization WH. Multisystem inflammatory syndrome in children and adolescents temporally related to COVID-19 (2020). Available from: https://www.who.int/news-room/commentaries/detail/multisystem-inflammatory-syndrome-in-children-and-adolescents-with-covid-19 (Accessed May 11, 2022).

[12] Panaro S Cattalini M. The Spectrum of manifestations of severe acute respiratory syndrome-coronavirus 2 (SARS-CoV2) infection in children: what we can learn from multisystem inflammatory syndrome in children (MIS-C). Front Med (Lausanne). (2021) 8:747190. 10.3389/fmed.2021.747190 34778310 PMC8581204

[13] Alkan G Sert A Oz SKT Emiroglu M Yılmaz R. Clinical features and outcome of MIS-C patients: an experience from Central Anatolia. Clin Rheumatol. (2021) 40(10):4179–89. 10.1007/s10067-021-05754-z 33956250 PMC8100744

[14] Sperotto F Friedman KG Son MBF VanderPluym CJ Newburger JW Dionne A. Cardiac manifestations in SARS-CoV-2-associated multisystem inflammatory syndrome in children: a comprehensive review and proposed clinical approach. Eur J Pediatr. (2021) 180(2):307–22. 10.1007/s00431-020-03766-6 32803422 PMC7429125

[15] Hoste L Van Paemel R Haerynck F. Multisystem inflammatory syndrome in children related to COVID-19: a systematic review. Eur J Pediatr. (2021) 180(7):2019–34. 10.1007/s00431-021-03993-5 33599835 PMC7890544

[16] Whittaker E Bamford A Kenny J Kaforou M Jones CE Shah P Clinical characteristics of 58 children with a pediatric inflammatory multisystem syndrome temporally associated with SARS-CoV-2. JAMA. (2020) 324(3):259–69. 10.1001/jama.2020.10369 32511692 PMC7281356

[17] Feldstein LR Rose EB Horwitz SM Collins JP Newhams MM Son MBF Multisystem inflammatory syndrome in U.S. Children and adolescents. N Engl J Med. (2020) 383(4):334–46. 10.1056/NEJMoa2021680 32598831 PMC7346765

[18] Mannarino S Raso I Garbin M Ghidoni E Corti C Goletto S Cardiac dysfunction in multisystem inflammatory syndrome in children: an Italian single-center study. Ital J Pediatr. (2022) 48(1):25. 10.1186/s13052-021-01189-z 35135600 PMC8822778

[19] Roarty C Waterfield T. Review and future directions for PIMS-TS (MIS-C). Arch Dis Child. (2022):archdischild-2021-323143. 10.1136/archdischild-2021-323143 35012934

[20] Glover HA Davis AB. A case of multisystem inflammatory syndrome in children following SARS-CoV-2 infection in a rural emergency department. Adv Emerg Nurs J. (2021) 43(2):114–22. 10.1097/TME.0000000000000346 33915560 PMC8098863

[21] Reiff DD Cron RQ. Who Would Have Predicted Multisystem Inflammatory Syndrome in Children? Current Rheumatology Reports. (2022). 10.1007/s11926-022-01056-8 PMC8852994 35150412

[22] Waseem M Shariff MA Tay ET Mortel D Savadkar S Lee H Multisystem inflammatory syndrome in children. J Emerg Med. (2022) 62(1):28–37. 10.1016/j.jemermed.2021.07.070 34538678 PMC8445772

[23] Vella LA Rowley AH. Current insights into the pathophysiology of multisystem inflammatory syndrome in children. Curr Pediatr Rep. (2021) 9:1–10. 10.1007/s40124-021-00257-6 34692237 PMC8524214

[24] Chou J Thomas PG Randolph AG. Immunology of SARS-CoV-2 infection in children. Nat Immunol. (2022) 23(2):177–85. 10.1038/s41590-021-01123-9 35105983 PMC8981222

[25] Sacco K Castagnoli R Vakkilainen S Liu C Delmonte OM Oguz C Immunopathological signatures in multisystem inflammatory syndrome in children and pediatric COVID-19. Nat Med. (2022) 28:1050–62. 10.1038/s41591-022-01724-3 35177862 PMC9119950

[26] KC S Awasthi P Kumar S Angurana SK Nallasamy K Angrup A MIS-C mimickers: a case series of bacterial enteritis and sepsis mistaken as MIS-C. Indian J Pediatr. (2022) 89(2):206. 10.1007/s12098-021-04019-6 34757575 PMC8579170

[27] Dworsky ZD Roberts JE Son MBF Tremoulet AH Newburger JW Burns JC. Mistaken MIS-C: a case series of bacterial enteritis mimicking MIS-C. Pediatr Infect Dis J. (2021) 40(4):e159–61. 10.1097/INF.0000000000003050 33710982 PMC7958995

[28] Kanis J Hall CL Pike J Kline JA. Diagnostic accuracy of the D-dimer in children. Arch Dis Child. (2018) 103(9):832–4. 10.1136/archdischild-2017-313315 29117965

[29] Acharya S Saxena A John BM Tewari VV. Atypical multisystem inflammatory syndrome in children (MIS-C). Indian J Pediatr. (2021) 88(7):715. 10.1007/s12098-021-03774-w 33931830 PMC8087532

[30] Han P Douillard J Cheng J Ramanathan A Tieu D Degner T. Multisystem inflammatory syndrome in children in a 15-year-old male with a retropharyngeal phlegmon. Case Rep Pediatr. (2020) 2020:6668371. 10.1155/2020/6668371 33274096 PMC7678743

[31] Nygaard U. Incidence of MIS-C Following SARS-CoV-2 Infection. Identifier: NCT05186597. Available from: https://clinicaltrials.gov/ct2/show/study/NCT05186597

[32] (NIAID) NIoAaID. COVID-19: Pediatric Research Immune Network on SARS-CoV-2 and MIS-C. Identifier: NCT04588363. Available from: https://clinicaltrials.gov/ct2/show/record/NCT04588363

[33] HealthCore-NERI. Long-Term Outcomes After the Multisystem Inflammatory Syndrome in Children (MUSIC). Available from: https://clinicaltrials.gov/ct2/show/record/NCT05287412

